# Inhibition of Radiation and Temozolomide-Induced Glioblastoma Invadopodia Activity Using Ion Channel Drugs

**DOI:** 10.3390/cancers12102888

**Published:** 2020-10-08

**Authors:** Marija Dinevska, Natalia Gazibegovic, Andrew P. Morokoff, Andrew H. Kaye, Katharine J. Drummond, Theo Mantamadiotis, Stanley S. Stylli

**Affiliations:** 1 Department of Surgery, The University of Melbourne, The Royal Melbourne Hospital, Parkville 3050, Victoria, Australia; mdinevska@student.unimelb.edu.au (M.D.); morokoff@unimelb.edu.au (A.P.M.); a.kaye@unimelb.edu.au (A.H.K.); kjd@unimelb.edu.au (K.J.D.); theo.mantamadiotis@unimelb.edu.au (T.M.); 2 Victoria University, St. Albans 3021, Victoria, Australia; natalia.gazibegovic@outlook.com; 3 Department of Neurosurgery, The Royal Melbourne Hospital, Parkville 3050, Victoria, Australia; 4 Hadassah University Medical Centre, Jerusalem 91120, Israel; 5 Department of Microbiology & Immunology, School of Biomedical Sciences, The University of Melbourne, Parkville 3010, Victoria, Australia

**Keywords:** glioma, glioblastoma, invasion, invadopodia, ion channels, drug repurposing

## Abstract

**Simple Summary:**

Glioblastoma accounts for approximately 40–50% of all primary brain cancers and is a highly aggressive cancer that rapidly disseminates within the surrounding normal brain. Dynamic actin-rich protrusions known as invadopodia facilitate this invasive process. Ion channels have also been linked to a pro-invasive phenotype and may contribute to facilitating invadopodia activity in cancer cells. The aim of our study was to screen ion channel-targeting drugs for their cytotoxic efficacy and potential anti-invadopodia properties in glioblastoma cells. We demonstrated that the targeting of ion channels in glioblastoma cells can lead to a reduction in invadopodia activity and protease secretion. Importantly, the candidate drugs exhibited a significant reduction in radiation and temozolomide-induced glioblastoma cell invadopodia activity. These findings support the proposed pro-invasive role of ion channels via invadopodia in glioblastoma, which may be ideal therapeutic targets for the treatment of glioblastoma patients.

**Abstract:**

Glioblastoma (GBM) is the most prevalent and malignant type of primary brain cancer. The rapid invasion and dissemination of tumor cells into the surrounding normal brain is a major driver of tumor recurrence, and long-term survival of GBM patients is extremely rare. Actin-rich cell membrane protrusions known as invadopodia can facilitate the highly invasive properties of GBM cells. Ion channels have been proposed to contribute to a pro-invasive phenotype in cancer cells and may also be involved in the invadopodia activity of GBM cells. GBM cell cytotoxicity screening of several ion channel drugs identified three drugs with potent cell killing efficacy: flunarizine dihydrochloride, econazole nitrate, and quinine hydrochloride dihydrate. These drugs demonstrated a reduction in GBM cell invadopodia activity and matrix metalloproteinase-2 (MMP-2) secretion. Importantly, the treatment of GBM cells with these drugs led to a significant reduction in radiation/temozolomide-induced invadopodia activity. The dual cytotoxic and anti-invasive efficacy of these agents merits further research into targeting ion channels to reduce GBM malignancy, with a potential for future clinical translation in combination with the standard therapy.

## 1. Introduction

Malignant brain tumors are among the most aggressive cancers, resulting in impaired health-related quality of life and survival measured in months or a few years [1]. As classified by the World Health Organization (WHO), glioblastoma (GBM) is a highly malignant grade IV astrocytoma accounting for approximately 50% of all gliomas [2,3,4]. Although GBM has an incidence of less than 10 per 100,000, it is incurable; thus, the burden of disease on patients and carers remains significant, resulting in an average loss of 12 years of life [5].

The current therapeutic regime for GBM patients follows the “Stupp Protocol”, which involves maximal safe surgical resection of the tumor followed by radiotherapy (RT) and concomitant chemotherapy with temozolomide (TMZ), an oral DNA alkylating agent and subsequent adjuvant TMZ for 6–12 months [6]. Despite a modest increase in survival since the introduction of TMZ (14.6 month median survival), 50% of GBM patients do not respond to TMZ, developing resistance to both RT and TMZ [7]. In addition, GBM widely infiltrates the surrounding brain parenchyma [8]. This invasive capacity hinders surgical resection, making gross tumor debulking impossible with inevitable tumor recurrence within 1–2 cm of the resection cavity [8,9]. 

Cancer cell invasion is a multi-step process orchestrated by tumor cell interactions with the tumor microenvironment [10]. This process is initiated by cell polarization and adhesion to the extracellular matrix (ECM), which is followed by acquired cancer cell mobility and ECM degradation [8]. More specifically, research has indicated that dynamic actin-rich subcellular protrusions known as invadopodia are integral in facilitating cancer cell invasion [11]. These structures serve to proteolytically degrade the ECM through the complex interactions within a network of signaling molecules and proteins [12,13]. Invadopodia can extend up to 8 µm into the surrounding environment and have a diameter ranging from 0.1 to 0.8 µm [12]. The physical force generated by actin polymerization and the action of transmembrane and secreted matrix metalloproteinases (MMP-2 and -9) in ECM degradation are integral to the invadopodia-mediated invasion of malignant cells [14]. Numerous proteins, including Tks5 (Tyrosine Kinase substrate with 5 SH3 domains) and cortactin, are involved in the processes required for the biogenesis of invadopodia, which include cell signaling, adhesion, and actin remodeling [15]. Our laboratory has previously demonstrated that expression levels of the invadopodia regulator Tks5 in human glioma biopsies are related to prognosis [16]. Importantly, the amplification of the cortactin gene (CTTN) is evident in a number of cancers, correlating with enhanced tumor invasiveness and poor prognosis [17,18]. 

Ion channels regulate several cancer promoting processes, including tumor cell invasion [19,20]. Under physiological conditions, potassium, sodium, and calcium channels are responsible for maintaining intracellular ionic balance, cell shape, and cell volume [21]. However, during tumorigenesis, the altered expression of ion channel genes can lead to the dysregulation of normal cellular functions, including proliferation, migration, and apoptosis [22]. For example, potassium channels, such as calcium-activated potassium channels (that is, KCa3.1), have been linked to enhanced invasion via the regulation of cellular ionic balance [19,23,24,25]. The gene encoding KCa3.1, KCNN4, is overexpressed in 32% of gliomas and correlates with shorter survival [26]. Additionally, potassium channels have been described to contribute to cancer cell invasion via their interaction with molecules associated with invadopodia formation, such as focal adhesion kinase (FAK), integrins, and cortactin [25]. Likewise, voltage-gated calcium channels (VGCCs) and non-voltage activated calcium permeable channels are associated with malignant transformations in a number of cancers, including glioma [27]. A study by Zhang et al. [28] demonstrated a decrease in cell migration in human GBM cell lines following the inhibition of T-type Ca^2+^ channels (low-voltage activated channels). Under resting membrane conditions, these ion channels play an integral role in the maintenance of intracellular Ca^2+^ and have been linked to tumor cell migration and invasion in GBM cells [29]. In the context of tumor cell invasion, the role of ion channels is only beginning to be understood [19]. The proposed influence of ion channels on actin cytoskeletal rearrangement and various proteins including cortactin and integrins may contribute to mechanisms mediating invadopodia formation and activity in cancer cells [30,31]. This highlights the potential for ion channel blockers to target invadopodia and inhibit GBM cell invasion. 

Over the past decade, the standard of care for GBM has remained unchanged, emphasizing the urgent need for drug discovery and development. This process requires high-throughput screening of candidate compounds, followed by extensive pre-clinical and clinical studies [32]. However, major limitations with this approach include both the time from the initial studies to clinical implementation (ranging from 11.4 to 13.5 years) and the associated costs ($161 million to $1.8 billion) [32]. For this reason, drug repurposing has become an increasingly attractive option. This is advantageous as it offers a reduction in the time required for pre-clinical and clinical studies and the associated costs, as drug toxicity, pharmacokinetics, dosage, and safety are already understood [33]. 

In this study, we investigated the cytotoxicity and anti-invasion activity of a panel of ion channel-binding drugs on GBM cells. The aim of this work was to screen a panel of 20 ion channel-targeting drugs for their ability to inhibit GBM cell viability and invadopodia activity. 

## 2. Results

### 2.1. GBM Tissue Exhibits Increased Invadopodia Regulator and Ion Channel Gene Expression

To investigate the clinical relevance of invadopodia regulator (Table 1) and ion channel (Table 2) genes in glioma, we used the online database, Oncomine^TM^. The expression levels of the pro-invadopodia regulators cortactin, MMP-2, Src, NWASP (Neural Wiskott-Aldrich syndrome protein), Tks4 (Tyrosine Kinase substrate with 4 SH3 domains), Tks5, and Nck (non-catalytic region of tyrosine kinase adaptor protein), and ion channel (calcium, sodium, and potassium) genes in GBM and non-tumor brain tissue were examined. MMP-2 and Nck1 (non-catalytic region of tyrosine kinase adaptor protein 1) were the most frequently overexpressed invadopodia regulators in GBM compared to non-tumor brain tissue (Table 1). 

The data presented in Table 2 demonstrate that the potassium and calcium ion channels, KCNH2 and CACNA1C, were most frequently overexpressed in GBM tissue compared to normal tissue. Subsequently, the SurvExpress online database for cancer gene expression data was used to examine the clinical relevance of invadopodia regulators and ion channel gene expression in GBM.

**Figure 1:**
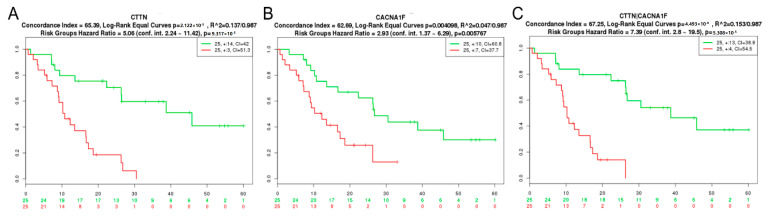
Co-expression of invadopodia regulator and ion channel genes correlates with poor GBM patient survival. (**A**) High invadopodia regulator (cortactin gene, CTTN), (**B**) ion channel (CACNA1S), and (**C**) combined gene expression indicates significantly poorer survival outcomes in GBM patients from the Nutt Louis dataset (deposited within the SurvExpress database). The Kaplan–Meier plots show the two risk groups, the log-rank test of differences between risk groups, the hazard ratio estimate, and the concordance indices.

Figure 1 demonstrates poorer survival outcomes for high gene expression of the pro-invadopodia regulator, CTTN, or ion channel, CACNA1F. The co-expression of CTTN and CACNA1F reveals a further impact on survival, and additional combinations are listed in Table 3. Together, these data suggests a role for ion channels in the process of glioma cell invasion mediated by invadopodia.

### 2.2. GBM Cells Form Functional Invadopodia and Express Invadopodia Regulator Proteins

**Figure 2:**
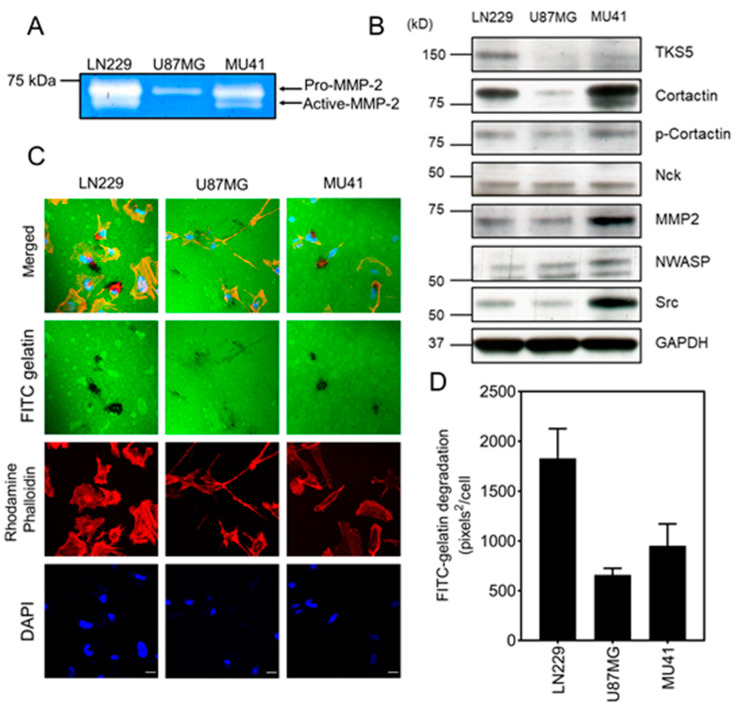
GBM cell lines secrete MMP-2 and form functional fluorescein isothiocyanate (FITC)–gelatin degrading invadopodia. (**A**) Gelatin-based zymogram analysis of LN229, U87MG, and MU41 cells cultured in serum-free conditioned media for 24 h showing MMP-2 activity. (**B**) Western blot analysis of GBM cell lines for a range of pro-invadopodia regulator proteins (Src, NWASP, MMP-2, Nck, phospho-cortactin, cortactin, and TKS5), the uncropped Western Blot figure is in Figure S2 (**C**) LN229, U87MG, and MU41 cells were plated on a thin film of cross-linked FITC-labeled gelatin for 24 h. Cells were stained with rhodamine phalloidin (red) to visualize actin filaments and DAPI (4′,6-diamidino-2-phenylindole) (blue) for cell nuclei. FITC–gelatin degradation is evident as black areas devoid of FITC-labeled gelatin. Images were acquired with a 60× oil immersion lens using a Nikon A1 confocal system. (**D**) Quantification of the basal invadopodia-mediated FITC–gelatin degradation. Representative of *n* = 3 independent experiments, error bars represent SEM. Scale bar = 20 µm.

Invadopodia are actin-rich protrusions that facilitate the invasion of tumor cells from the tumor bulk into the surrounding healthy parenchyma [12,13]. MMPs (specifically MMP-2 and MMP-9) are enriched and secreted at the tips of invadopodia, thus mediating the proteolytic degradation of the ECM [11]. We utilized gelatin-based zymography to examine the conditioned medium isolated from three GBM cell lines and determine the extracellular secreted levels of MMP-2 and MMP-9. Analysis of the conditioned media of the LN229, U87MG, and MU41 GBM cell lines revealed the presence of pro-MMP-2 (72 kDa) and active-MMP-2 (65 kDa). The LN229 cell line displayed the highest level of MMP-2 secretion, while the U87MG cell line showed the least (Figure 2A). 

Several invadopodia regulator proteins, including Tks5, Src, and cortactin, participate in the formation and matrix-degrading activity of invadopodia. Therefore, we next examined the expression of these proteins in the LN229, U87MG, and MU41 cell lines (Figure 2B). The protein expression profile of these regulators varied across cell lines, with Tks5 highly expressed in LN229 cells. Cortactin was highly expressed in the MU41 and LN229 cell lines. Notably, Src, MMP-2, and NWASP were detected at higher levels in the MU41 cell line.

As the GBM cells secreted MMP-2 and expressed a range of invadopodia regulator proteins, we next used a fluorescent gelatin matrix degradation assay to determine whether the GBM cell lines could form functional invadopodia. This assay, which measures MMP-2-mediated invasion by determining the clearance of fluorescein isothiocyanate (FITC)-labeled gelatin (absence of green) colocalized with rhodamine phalloidin actin-stained puncta, showed activity in the three GBM cell lines (Figure 2C). LN229 cells exhibited the highest FITC–gelatin degrading activity, followed by MU41 and U87MG cells (Figure 2D). This observation was consistent with the levels of MMP-2 secreted by each cell line (Figure 2A).

**Figure 3:**
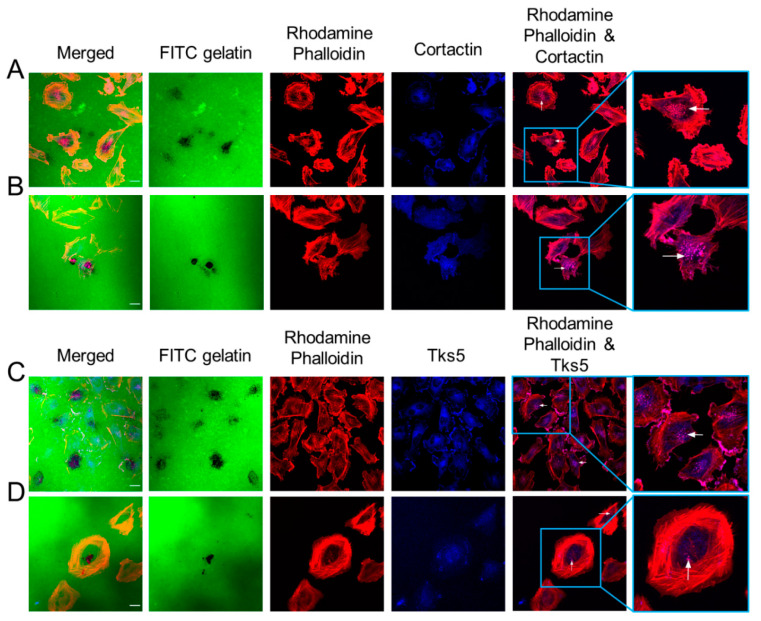
Cortactin and Tks5 co-localize with actin in GBM cell invadopodia. LN229 and MU41cells were seeded on FITC-labeled gelatin coverslips for 24 h prior to fixing and staining for rhodamine phalloidin to probe for F-actin filaments, cortactin, and Tks5 primary antibodies and an Alexa 405 secondary antibody (blue). Images were acquired with a 60× oil immersion lens using a Nikon A1 confocal system. Images displayed co-localization with actin puncta as follows: LN229 ((**A**)-cortactin), MU41 ((**B**)-cortactin), LN229 ((**C**)-Tks5), and MU41 ((**D**)-Tks5). White arrows denote co-localization with cortactin or Tks5 and actin puncta. Figure is representative of *n* = 3 experiments. Scale bar = 20 µm.

To further verify the presence of invadopodia, we co-stained GBM cells for Tks5 and cortactin as they colocalize with invadopodia and are integral for invadopodia formation and activity. As demonstrated in Figure 3, the co-localization of Tks5/cortactin with actin puncta validated the presence of invadopodia in the LN229, U87MG, and MU41 GBM cell lines.

### 2.3. Ion Channel-Targeting Drugs Reduce GBM Cell Viability

The current standard of care for GBM patients is inadequate, and there is an urgent need to develop additional treatment options to target tumor cells that survive radiotherapy and chemotherapy. To identify ion channel-binding drugs that can kill GBM cells, we screened 20 drugs (Table 4) that inhibit ion channels (It must be noted that flunarizine dihydrochloride is the only ion channel drug in the list that is not currently FDA (Food and Drug Administration)-approved for clinical use in the USA). The drugs were first screened for their ability to reduce GBM cell viability using an MTT (3-(4,5-dimethylthiazol-2-yl)-2,5-diphenyltetrazolium bromide) cell proliferation assay and then screened for their anti-invasive properties and regulation of invadopodia activity. The cytotoxicity of the 20 ion channel drugs was assessed across a range of concentrations (0.01, 0.1, 1, and 10 μM) in the three GBM cell lines (Figure S1).

### 2.4. Ion Channel Drugs Reduce MMP-2 Secretion and Invasion

**Figure 4:**
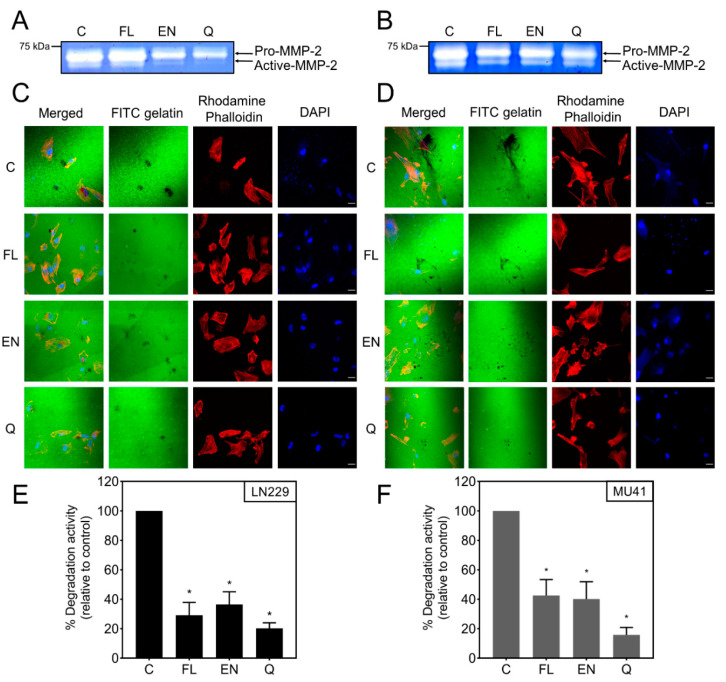
Ion channel drugs inhibit MMP-2 secretion and invadopodia activity in GBM cells. Gelatin-based zymogram analysis of (**A**) LN229 and (**B**) MU41 GBM cells in serum-free conditioned media treated with 10 µM flunarizine dihydrochloride (FL), econazole nitrate (EN), and quinine hydrochloride dihydrate (Q) for 72 h. Representative of *n* = 2 experiments. (**C**) LN229 and (**D**) MU41 GBM cells were seeded on coverslips coated with a thin film of cross-linked FITC-labeled gelatin following 72-h treatment with 10 µM of FL, EN, and Q. Following a 24-h incubation, cells were fixed and stained with rhodamine phalloidin (red) to probe for actin filaments and DAPI (blue) for nuclear staining. Black areas devoid of FITC-labeled gelatin represent areas of gelatin degradation. Graphical representation of (**E**) LN229 and (**F**) MU41 invadopodia mediated FITC–gelatin degradative activity (relative to untreated control). Mean of *n* = 3 independent experiments, error bars represent SEM, * *p* < 0.05. Scale bar = 20 µm.

Following the shortlisting of ion channel drugs based on their cytotoxicity profiles, we examined the impact of the drugs on MMP-2 secretion. LN229 and MU41 cells were investigated further, as they had the highest MMP-2 secretion and invadopodia activity (Figure 2). Econazole nitrate and quinine hydrochloride dihydrate-treated LN229 and MU41 cells showed a decrease in MMP-2 secretion, while flunarizine dihydrochloride led to a reduction of MMP-2 secretion in MU41 cells only (Figure 4A,B). Subsequently, we investigated the ability of these drugs to reduce invadopodia-mediated FITC–gelatin degradation (Figure 4C–F). All three drugs resulted in reduced invadopodia-mediated gelatin-degradation activity in both GBM cells lines. Quinine hydrochloride dihydrate treatment resulted in the greatest reduction in FITC–gelatin degradation in both cell lines. This is consistent with the reduced level of MMP-2 secretion following quinine hydrochloride dihydrate treatment. Interestingly, the level of MMP-2 secretion in flunarizine dihydrochloride-treated LN229 cells (Figure 4A) did not correspond to the inhibitory effect seen in the invadopodia-mediated FITC–gelatin degradation assay, indicating that this drug may influence other factors regulating invadopodia biogenesis.

### 2.5. MMP-2 Secretion and Invadopodia Gelatin Degradation Is Enhanced Following Radiation and Temozolomide Treatment

**Figure 5:**
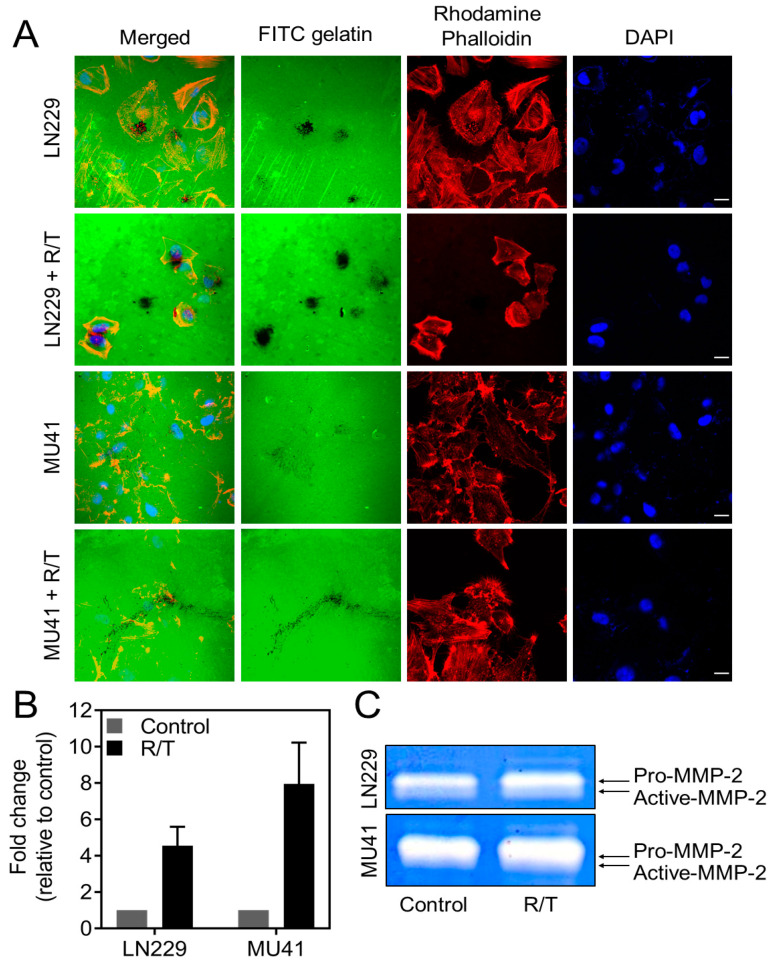
Radiation/temozolomide (R/T) treatment enhances MMP-2 secretion and invadopodia activity in GBM cells (**A**) LN229 and MU41 GBM cells were seeded and incubated for 24 h on coverslips coated with a thin film of cross-linked FITC-labeled gelatin 24 h post-treatment with R/T (2 Gy/50 µM). Then, the cells were stained with rhodamine–phalloidin (red) for actin filaments and DAPI for nuclei labelling (blue). FITC–gelatin degradation is evident as black areas devoid of FITC-labeled gelatin. (**B**) Fold change of FITC–gelatin degradation per GBM cell relative to the corresponding untreated cells for each cell line. Mean of *n* = 3 independent experiments, error bars represent SEM. Scale bar = 20 µm, applicable to all images in the panel. (**C**) LN229 and MU41 GBM cells were treated with R/T (2 Gy/50 µM) for 24 h and incubated in serum-free Optimem before conditioned medium was analyzed via gelatin-based zymography. Representative of *n* = 2 independent experiments.

To investigate the impact of RT and TMZ on invadopodia-mediated invasion, GBM cells were treated with 2 Gy RT and 50 μM TMZ. Zymographic analysis of conditioned GBM cell medium highlighted an increase in pro-MMP-2 secretion in both LN229 and MU41 cell lines, while an increase in active-MMP-2 was seen in the LN229 cell line only (Figure 5C). As MMP-2 secretion increased following RT/TMZ treatment, we next examined the effect of RT/TMZ treatment on the ability of GBM cells to form functional FITC–gelatin-degrading invadopodia. The GBM cell lines showed an increase in the level of invadopodia-mediated FITC–gelatin degradation post-RT/TMZ treatment (Figure 5A,B). The increase in MMP-2 secretion and invadopodia-mediated FITC–gelatin degradation suggests that cells that survive RT/TMZ treatment may acquire a more pro-invasive phenotype.

### 2.6. Inhibition of RT/TMZ-Induced Invadopodia Activity

**Figure 6:**
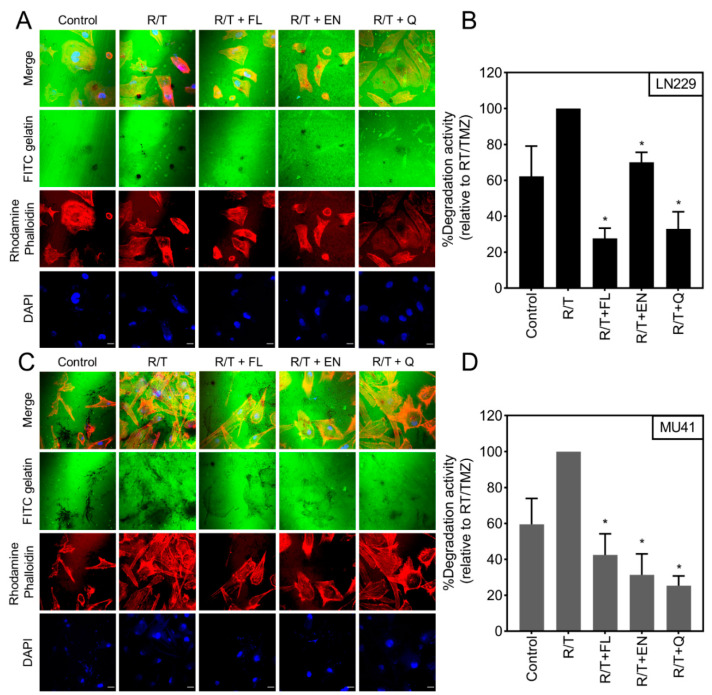
Ion channel-targeting drugs reduce radiation and temozolomide enhanced invadopodia activity. (**A**) LN229 and (**B**) MU41 GBM cells were subjected to R/T (2 Gy/50 µM) and 10 µM treatment of flunarizine dihydrochloride (FL), econazole nitrate (EN), or quinine hydrochloride dihydrate (Q) for 72 h prior to being seeded a thin film of cross-linked FITC-labeled gelatin. Cells were stained with rhodamine phalloidin (red) to visualize actin filaments and DAPI (blue) for cell nuclei. FITC–gelatin degradation is evident as black areas devoid of FITC-labeled gelatin. Graphical representation of (**C**) LN229 and (**D**) MU41 invadopodia-mediated FITC–gelatin degradative activity per GBM cell (relative to R/T treated groups). Mean of *n* = 3 independent experiments, error bars represent SEM, * *p* < 0.05. Scale bar = 20 µm.

We next examined the ability of the selected candidate drugs to reduce RT/TMZ-induced invadopodia activity in the GBM cells. Here, we demonstrate that treatment with flunarizine dihydrochloride, econazole nitrate, and quinine hydrochloride dihydrate resulted in a significant decrease in RT/TMZ induced invadopodia activity in both the LN229 and MU41 GBM cells (Figure 6). The greatest reduction in invadopodia-mediated FITC–gelatin degradation was noted with quinine hydrochloride dihydrate in the MU41 cell line and flunarizine dihydrochloride in the LN229 cell line.

**Figure 7:**
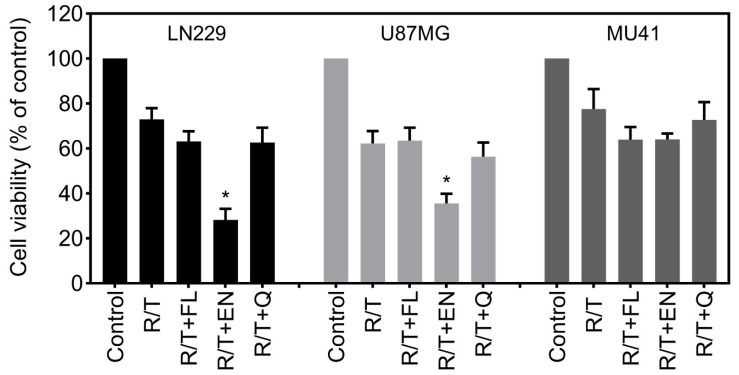
Econazole nitrate can further reduce cell viability following R/T treatment. LN229, U87MG and MU41 GBM cells were treated R/T (2 Gy/50 µM) and 10 μM of each candidate drug: flunarizine dihydrochloride (FL), econazole nitrate (EN), and quinine hydrochloride dihydrate (Q) for 7 days. Subsequently, cell viability was determined using an MTT (3-(4,5-dimethylthiazol-2-yl)-2,5-diphenyltetrazolium bromide) cell proliferation assay. Cell viability is represented as a percentage relative to the control cells. Mean of *n* = 3 experiments, error bars represent SEM, * *p* < 0.05 (relative to R/T).

With a focus on identifying drugs with dual “anti-invasive” and “cytotoxic” activity on RT/TMZ-resistant GBM cells, we also examined cell viability with the three candidate drugs in RT/TMZ pre-treated cells. While these drugs led to a reduction in cell viability in most cell lines when compared to the RT/TMZ-only treated, only econazole nitrate in conjunction with RT/TMZ significantly reduced the cell viability of LN229 and U87MG cells when compared to RT/TMZ-only treated cells (Figure 7).

## 3. Discussion

Despite a multi-modal treatment approach involving maximal-safe surgical resection, adjuvant radiotherapy, and chemotherapy, GBM remains invariably fatal, which is facilitated by infiltrative growth into the surrounding healthy brain tissue [44]. Tumor cell invasion is a complex process involving cancer cell cytoskeletal changes and remodeling of the ECM mediated by invadopodia [14,45,46,47]. Ion channels have been shown to facilitate various aspects of cancer progression, including invasion [48]. Koltai [49] has proposed a role for sodium ion channels in protease secretion, while potassium channels can contribute to an invasive phenotype through the altered regulation of intracellular Ca^2+^ levels, leading to cytoskeletal changes [19,49]. Importantly, the relevance of invadopodia and ion channels in GBM can be highlighted by the overexpression of key invadopodia regulators (Table 1) and ion channels in GBM tissue (Table 2) and their impact on glioma patient survival (Figure 1).

The aim of this work was to screen a panel of 20 ion channel-targeting drugs for their ability to select candidate drugs that inhibit GBM cell viability and invadopodia activity. This study identified three drugs that behaved in a “dualistic” manner, reducing both cell viability and invadopodia activity. The shortlisted drugs were flunarizine dihydrochloride (a calcium antagonist approved for use for the prevention of migraines) [50,51], econazole nitrate (an anti-fungal calcium antagonist) [52], and quinine hydrochloride dihydrate (an anti-malarial potassium channel blocker) [53]. A decrease in MMP-2 secretion (Figure 4A,B) was observed with econazole nitrate and quinine hydrochloride dihydrate in both the LN229 and MU41 GBM cell lines, while flunarizine dihydrochloride resulted in reduced MMP-2 secretion only in the MU41 cell line.

Furthermore, treatment with the three shortlisted ion channel drugs revealed a statistically significant reduction in the level of invadopodia-mediated FITC–gelatin degradation activity in the GBM cell lines (Figure 4C–F), suggesting that all three drugs exhibit an “anti-invadopodia” effect. These findings were consistent with the trend seen in the reduced level of MMP-2 secretion following treatment with econazole nitrate and quinine hydrochloride dihydrate (Figure 4A,B). This w–s not observed with flunarizine dihydrochloride treatment of the LN229 GBM cell line. Although flunarizine dihydrochloride led to a decrease in invadopodia-mediated FITC–gelatin degradation, the secreted levels of MMP-2 were not significantly altered, indicating that flunarizine dihydrochloride may exert its action on the dynamics of actin cytoskeletal reorganization and subsequent invadopodia formation and not on the secretion of proteases (Figure 4A). Studies have proposed that Ca^2+^ oscillations are required for the initiation of invadopodia formation [48,54]. Flunarizine dihydrochloride, as a calcium channel antagonist, may reduce Ca^2+^ oscillations and interfere with the assembly of invadopodia. An impact on the dynamics of invadopodia could lead to a reduction in the number and size of invadopodia (less invadopodia/smaller invadopodia leading to reduced focal FITC–gelatin degradation) [55,56].

Several studies have shown that radiotherapy promotes an invasive and pro-migratory phenotype in GBM [57,58]. Trog et al. [59] showed that RT and TMZ promote the upregulation of pro-invasive proteins such as MMP-2 and MT1-MMP (Membrane type 1-matrix metalloproteinase) in vitro. Consistent with these findings, we demonstrated that clinically relevant doses of RT and TMZ not only led to an increase in the level of MMP-2 secretion but also an increase in invadopodia-mediated FITC–gelatin degradation activity (Figure 5) in GBM cells. Such findings suggest that RT/TMZ treatment promotes an invasive phenotype through invadopodia via the upregulated expression and secretion of MMP-2 in cells that survive treatment [59].

Considering the increase in invasion following RT and TMZ treatment and the “anti-invadopodia” activity of the ion channel drugs, we sought to examine whether treatment with these agents could inhibit treatment-induced enhanced invadopodia activity. While econazole nitrate was the only drug to result in a significant reduction in cell viability following RT/TMZ treatment (Figure 7), all three drugs led to a significant reduction in RT/TMZ-induced invadopodia activity (Figure 6). These results suggest that targeting ion channels can overcome the infiltrative and invasive properties of GBM cells by interfering with invadopodia activity. Flunarizine dihydrochloride and econazole nitrate both block calcium channels and may promote anti-invasive activity. This may occur via the inhibition of the Ca^2+^ influx required for the activation of other ion channels, such as potassium and chloride channels, which are known to enhance cell migration and invasion [60]. Alternatively, these agents may also act to reduce the invadopodia-mediated focal ECM degradation by blocking the Ca^2+^ signaling required for the upregulation of proteolytic enzymes such as MMPs and cathepsins [54,60]. As a potassium channel blocker, quinine hydrochloride dihydrate may act by antagonizing potassium channels such as KCa3.1. These channels are involved in mediating cell volume changes required for the reorganization of the actin cytoskeleton and promoting an invasive phenotype in cancer cells [23,24,61].

After identifying drugs that complement the current recommended therapy for GBM patients, one must also consider drug delivery and blood–brain barrier (BBB) penetrance. This is a significant challenge in the use of small molecule compounds in the clinical treatment of GBM where the penetrance of the BBB is limited [62]. Flunarizine dihydrochloride has been shown to have a concentration in the brain that is 10 times higher than in the plasma [50,51,63], whilst quinine has been used for the treatment of malarial-causing parasites in the central nervous system [53,64]. Furthermore, econazole nitrate has also been patented as a neuroprotective agent (US 2010/0298394A1) [52]. This suggests that these drugs can cross the BBB. The predicted BBB penetrance of the candidate ion channel drugs is presented in Table S2. Plasma levels that have been achieved for the candidate agents are listed in Table S3. Therefore, we propose that flunarizine dihydrochloride, econazole nitrate, and quinine hydrochloride dihydrate demonstrate the potential for clinical applicability as an adjuvant treatment, in combination with the current standard of care for GBM patients. Furthermore, we posit that targeting ion channels to regulate invadopodia activity and inhibit GBM cell invasion is a promising therapeutic avenue that merits further investigation.

## 4. Materials and Methods

### 4.1. Ion Channel Drugs

The ion channel drugs used in this study were supplied by Selleckchem, (Selleckchem, Houston, TX, USA) at a concentration of 10 mM in DMSO and were stored at −80 °C until use. The ion channel drugs utilized in this study were included within a larger commercial library supplied by Selleckchem and were screened to provide preliminary data examining their impact on invadopodia activity in GBM cells, as there is evidence that ion channels can regulate tumor cell invasion [19,20].

### 4.2. Cell Lines and Cell Culture

LN229, U87MG, and MU41 human GBM cell lines were maintained in Dulbecco’s Modified Eagle’s Medium (Life Technologies, Carlsbad, CA, USA) supplemented with 10% heat-inactivated fetal bovine serum (HyClone, Global Life Sciences Solutions, Parramatta, Australia) and 1% antibiotic-antimycotic (Life Technologies). All cell lines were incubated at 37 °C in a 10% CO2 humidified atmosphere. LN229 and U87MG human GBM cell lines were obtained from the ATCC Biological material repository. The MU41 human GBM cell line was harvested from a GBM patient biopsy sample at the Royal Melbourne Hospital (Melbourne Health Research Ethics Approval Number HREC 2009.116).

### 4.3. Zymographic Analysis

LN229 and MU41 GBM cells were seeded in 6-well plates and allowed to adhere for 24 h. Cells were washed with sterile phosphate-buffered saline (PBS) before a further 24-h incubation in 2 mL serum-free Optimem^®^ (Thermo Fisher Scientific, Waltham, MA, USA). Then, 200 μL aliquots of the conditioned Optimem^®^ medium were sampled and stored at −80 °C until further use. Optimem^®^ media samples were normalized based on the GBM cell protein concentration using a BCA (bicinchoninic acid) protein assay (Pierce, ThermoFisher Scientific) and by performing densitometry of GAPDH bands in corresponding Western blots. The conditioned medium samples were diluted 1:1 with 2× Novex tris-glycine SDS sample buffer (Invitrogen, Thermo Fisher Scientific, Waltham, MA, USA) prior to being loaded in 10% gelatin substrate zymogram NuPAGE 10 well pre-cast gels (Novex, Invitrogen, Thermo Fisher Scientific, Waltham, MA, USA) and separated at 125 V for 90 min in 1× Novex tris-glycine SDS running buffer. The gels were incubated in 1× Novex zymogram renaturing buffer for 30 min and subsequently incubated in 1× Novex zymogram developing buffer for 30 min. Then, they were incubated overnight at 37 °C with fresh 1× Novex zymogram developing buffer. Following incubation, the gels were washed in distilled water before being stained in SimplyBlue SafeStain (Life Technologies) for 1 h. The gels were washed with distilled water until clear bands (representing gelatinolytic activity) against the undigested blue-stained gel were visible; then, gels were scanned using a flatbed scanner, and image files were used for analysis.

### 4.4. Invadopodia Degradation Assay

Fluorescein isothiocyanate (FITC)-conjugated gelatin prepared as per previously established protocol [65] was used to coat 25 mm round coverslips. LN229 and MU41 GBM cells were pre-treated with either 2 Gy radiation and 50 μM of temozolomide, or 10 μM of FL, EN, and Q. Following a 72-h incubation, cells were trypsinised and seeded at a density of 1 × 10^5^ cells per FITC-conjugated gelatin coverslip and incubated for a further 24 h. The cells were washed with PBS, fixed in 4% paraformaldehyde, and then permeabilized using with 0.2% Triton-X-100. Subsequently, they were stained with rhodamine phalloidin (actin filaments and invadopodia puncta) and DAPI (nuclei), and the coverslips mounted on glass slides with VectaShield (Vector Laboratories, Burlingame, CA, USA) mounting medium. Images were acquired using a Nikon A1+ confocal microscope system with a Plan Apo VC 60× Oil DIC N2 immersion objective. A total of 10–15 images were acquired for each experimental condition. Image J (Version 1.52e) was used for the analysis of the confocal images. The region and threshold tools were used to define the total area of FITC–gelatin degradation in an image field, while the particle counter macro was used to determine the area of degradation, which was normalized with respect to the number of cells (DAPI-positive nuclei).

### 4.5. Cell Viability

LN229, U87MG, and MU41 GBM cells were seeded (1 × 10^4^ cells /100μl) in 96-well plates and allowed to adhere for 24 h. Initially, the cells were treated with the 20 ion channel drugs over a concentration range (0.01, 0.1, 1, and 10 μM) (Table 1) for 7 days. Further experiments examining the effect of the shortlisted ion channel agents on cell viability in conjunction with RT and TMZ involved 2 Gy RT and 50 μM of TMZ pre-treatment 4 h prior to the addition of the ion channel drugs (10 μM). A CellTiter 96 Non-Radioactive Cell Proliferation Assay (MTT) (Promega, Alexandria, Australia) was used as per the manufacturer’s instructions to assess cell viability post-treatment.

### 4.6. Western Blot Analysis

Western blot analysis of GBM cell protein lysates (20 μg) was performed using NuPAGE 4–12% Bis-Tris pre-cast gels (Invitrogen) and transferred onto a 0.45 μm nitrocellulose blotting membrane (GE Healthsciences, Parramatta, Australia). The membrane was blocked in 3% bovine serum albumin in 1% TBST (Tris-buffered saline with Tween) for 1 h prior to overnight incubation with a primary antibody, including GAPDH, NWASP, Nck-1, phospho-cortactin (1:1000, Cell Signalling Technologies, Danvers, MA, USA), MMP-2, c-Src, cortactin, and Tks5 (1:1000, Santa Cruz Biotechnology). The membrane was subsequently incubated with the appropriate secondary antibody and developed using enhanced chemiluminescence reagent (GE Healthcare, Melbourne, Australia) and exposure onto Fujifilm Super RX film.

### 4.7. Oncomine Data Mining

Differential mRNA and DNA expression levels of invadopodia regulators and ion channels in GBM tissue were retrieved from the Oncomine^TM^ v4.5 (www.oncomine.org Compendia Bioscience™, Ann Arbor, MI, USA, part of Life Technologies) database. Oncomine^TM^ is an online cancer microarray database containing 715 datasets (86,733 samples) compiled from various studies. The threshold for inclusion for data analysis was set to *p* < 0.05 for significance and an mRNA expression fold difference of >2. All data are log transformed, and the standard deviation is normalized to one per array studied. A list of all analyzed genes is provided in Table S1. Further details regarding the Oncomine^TM^ analyses are provided in the Supplementary Methods.

### 4.8. SurvExpress

Glioma patient survival analysis was conducted using gene expression datasets deposited in the SurvExpress (http://bioinformatica.mty.itesm.mx/SurvExpress) database [66]. SurvExpress is an online database for evaluating cancer gene expression data using survival analysis. Data sourced from the SurvExpress platform was used for the survival analysis of invadopodia regulator and ion channel gene co-expression in glioma patients. Further details regarding the SurvExpress analyses are provided in the Supplementary Methods.

### 4.9. Statistical Analysis

Statistical significance was determined using an unpaired, unequal variance, two-tailed t-test with the use of GraphPad Prism 7 (Prism 7.00 for Windows, GraphPad Software, La Jolla, CA, USA, www.graphpad.com). Values were considered statistically significant if the *p* < 0.05. In all figures * denotes *p* < 0.05 and error bars represent the standard error of the mean (SEM).

## 5. Conclusions

During recent years, studies have been investigating the role of invadopodia in mediating cancer invasion. GBM, the most common primary brain tumor, is a highly proliferative and invasive cancer, and the current standard therapy involving surgical resection, radiotherapy, and chemotherapy (temozolomide) is insufficient to eradicate the tumor. Therefore, new therapies are required to not only reduce the number of cells surviving the current therapy but also to reduce the neurologically destructive invasive ability of the GBM cells. Ion channels have emerged as contributors to tumor pathophysiology in the various hallmarks of cancer including cell proliferation, migration, and invasion via their capacity in cell volume regulation. The data from our current study demonstrates that there is potential for repurposing ion channel agents with ion channel targets as novel prospective therapeutic agents to be utilized in targeting the invasive GBM cells that survive the current treatment for GBM patients.

## Figures and Tables

**Table 1:** Invadopodia regulator genes are overexpressed in glioblastoma (GBM) tissue compared to normal brain tissue.

Invadopodia Marker	Number of GBM Tissue Samples	Number of Normal Tissue Samples	Total Measured Genes	Mean Fold Change (Log2)	*p* Value	Sample Type	Platform	Study
CTTN	542	10	12,624	1.353	3 × 10^−3^	mRNA	Human Genome U2A	TCGA
MMP2	27	4	14,836	6.426	5.00 × 10^−4^	mRNA	ND	Bredel Brain 2 [34]
MMP2	30	3	9957	4.537	3.00 × 10^−3^	mRNA	ND	Liang [35]
MMP2	80	4	19,574	2.92	2.98 × 10^−4^	mRNA	Human Genome U2A	Murat [36]
MMP2	81	23	19,574	3.548	7.99 × 10^−16^	mRNA	Human Genome U2A	Sun [37]
MMP2	542	10	12,624	4.818	4.06 × 10^−10^	mRNA	Human Genome U2A	TCGA
Nck1	27	4	14,836	1.717	1.00 × 10^−2^	mRNA	ND	Bredel Brain 2 [34]
Nck1	30	3	9957	1.626	1.90 × 10^−2^	mRNA	ND	Liang [35]
Nck1	80	4	19,574	1.885	5.00 × 10^−3^	mRNA	Human Genome U2A	Murat [36]
Nck1	81	23	19,574	1.305	5.41 × 10^−7^	mRNA	Human Genome U2A	Sun [37]
Nck1	542	10	12,624	2.056	4.06 × 10^−9^	mRNA	Human Genome U2A	TCGA
Nck2	80	4	19,574	1.135	2.00 × 10^−3^	mRNA	Human Genome U2A	Murat [36]
NWASP	81	23	19,574	1.338	1.10 × 10^−2^	mRNA	Human Genome U2A	Sun [37]
Src	80	4	19,574	1.035	4.50 × 10^−2^	mRNA	Human Genome U2A	Murat [36]
Src	81	23	19,574	1.601	2.00 × 10^−3^	mRNA	Human Genome U2A	Sun [37]
Tks4	27	4	14,836	3.257	1.12 × 10^−4^	mRNA	ND	Bredel Brain 2 [34]
Tks4	30	3	9957	1.492	1.40 × 10^−2^	mRNA	ND	Liang [35]
Tks4	80	4	19,574	2.241	1.32 × 10^−6^	mRNA	Human Genome U2A	Murat [36]
Tks4	81	23	19,574	2.194	2.50 × 10^−4^	mRNA	Human Genome U2A	Sun [37]
Tks5	27	4	14,836	1.399	4.80 × 10^−2^	mRNA	ND	Bredel Brain 2 [34]
Tks5	22	7	7689	1.263	5.00 × 10^−3^	mRNA	ND	Yamanaka [38]

mRNA expression levels of invadopodia regulators in GBM and normal brain tissue were retrieved from the Oncomine database (mean fold change versus normal brain and overall *p* value in that dataset are displayed. The Student t-test is used to generate the *p* value). Gene expression data have been log transformed and normalized. Not defined (ND); Human Genome U2A—Human Genome U133 Plus 2.0 Array, TCGA—The Cancer Genome Atlas; MMP2—matrix metalloproteinase-2; NWASP—Neural Wiskott–Aldrich syndrome protein; Tks4—tyrosine kinase substrate with 4 SH3 domains; Tks5—tyrosine kinase substrate with 5 SH3 domains; Nck—non-catalytic region of tyrosine kinase adaptor protein; mRNA—messenger RNA; RNA—ribonucleic acid.

**Table 2:** Ion channel genes are overexpressed in GBM tissue compared to normal brain tissue.

Ion Channel Gene	Number of GBM Tissue Samples	Number of Normal Tissue Samples	Total Measured Genes	Mean Fold Change (Log2)	*p* Value	Sample Type	Platform	Study
KCNH2	106	32	18,823	1.175	3.49 × 10^−13^	DNA	RefSeq Genes	Beroukhim Brain [39]
KCNA5	107	32	18,823	1.054	2.15 × 10^−4^	DNA	RefSeq Genes	Beroukhim Brain [39]
KCNJ10	107	33	18,823	1.041	2.00 × 10^−3^	DNA	RefSeq Genes	Beroukhim Brain [39]
KCNB1	107	33	18,823	1.064	2.00 × 10^−3^	DNA	RefSeq Genes	Beroukhim Brain [39]
CACNA1S	107	33	18,823	1.041	3.20 × 10^−2^	DNA	RefSeq Genes	Beroukhim Brain [39]
CACNA1C	107	33	18,823	1.041	1.20 × 10^−2^	DNA	RefSeq Genes	Beroukhim Brain [39]
KCNN4	21	3	14,836	2.219	1.40 × 10^−2^	mRNA	ND	Bredel Brain 2 [34]
CACNA1D	27	4	14,836	1.377	6.00 × 10^−3^	mRNA	ND	Bredel Brain 2 [34]
CACNA1C	22	3	19,574	8.62	6.00 × 10^−3^	mRNA	Human Genome U2A	Lee Brain [40]
KCNH2	22	3	19,574	1.869	1.00 × 10^−3^	mRNA	Human Genome U2A	Lee Brain [40]
KCNB1	22	3	19,574	2.325	2.10 × 10^−2^	mRNA	Human Genome U2A	Lee Brain [40]
CACNA1D	22	3	19,574	3.293	3.85 × 10^−4^	mRNA	Human Genome U2A	Lee Brain [40]
KCNH2	80	4	19,574	1.252	5.00 × 10^−3^	mRNA	Human Genome U2A	Murat Brain [36]
KCNH2	27	7	8603	1.101	1.20 × 10^−2^	mRNA	Human Genome U95A	Shai Brain [41]
KCNH2	81	23	19,574	2.142	1.70 × 10^−2^	mRNA	Human Genome U2A	Sun Brain [37]
KCNH2	542	10	12,624	1.094	1.20 × 10^−2^	mRNA	Human Genome U2A	TCGA
KCNH2	582	37	18,823	1.320	4.80 × 10^−169^	DNA	RefSeq Genes	TCGA 2
KCNA5	582	37	18,823	1.034	5.75 × 10^−8^	DNA	RefSeq Genes	TCGA 2
KCNJ10	582	37	18,823	1.061	1.65 × 10^−46^	DNA	RefSeq Genes	TCGA 2
KCNB1	582	37	18,823	1.126	3.54 × 10^−68^	DNA	RefSeq Genes	TCGA 2
KCNN4	582	37	18,823	1.046	6.02 × 10^−10^	DNA	RefSeq Genes	TCGA 2
SCN5A	582	37	18,823	1.019	6.64 × 10^−5^	DNA	RefSeq Genes	TCGA 2
SCN8A	582	37	18,823	1.012	1.60 × 10^−2^	DNA	RefSeq Genes	TCGA 2
CACNA1S	582	37	18,823	1.055	2.28 × 10^−30^	DNA	RefSeq Genes	TCGA 2
CACNA1C	582	37	18,823	1.032	1.11 × 10^−7^	DNA	RefSeq Genes	TCGA 2
CACNA1D	582	37	18,823	1.013	7.00 × 10^−3^	DNA	RefSeq Genes	TCGA 2
CACNA1B	582	37	18,823	1.019	1.00 × 10^−3^	DNA	RefSeq Genes	TCGA 2
CACNA1G	582	37	18,823	1.027	4.56 × 10^−16^	DNA	RefSeq Genes	TCGA 2

mRNA and DNA expression levels of ion channels in GBM and normal brain tissue were retrieved from the Oncomine database (mean fold change versus normal brain and overall *p* value in that dataset are displayed. The Student t-test is used to generate the *p* value.). Gene expression data have been log transformed and normalized. Not defined (ND); Human Genome U2A—Human Genome U133 Plus 2.0 Array; Human Genome U95A—Human Genome U95A-Av2 Array; DNA—deoxyribonucleic acid; mRNA—messenger RNA; RNA—ribonucleic acid; TCGA—The Cancer Genome Atlas; KCNH2—hERG (the human Ether-à-go-go-Related Gene); KCNA5—Potassium voltage-gated channel, shaker-related subfamily, member 5; KCNJ10—Potassium Inwardly Rectifying Channel Subfamily J Member 10; KCNB1—Potassium Voltage-Gated Channel Subfamily B Member 1; CACNA1S—Calcium Voltage-Gated Channel Subunit Alpha1 S; CACNA1C—Calcium Voltage-Gated Channel Subunit Alpha1 C; KCNN4—Potassium Calcium-Activated Channel Subfamily N Member 4; CACNA1D—Calcium Voltage-Gated Channel Subunit Alpha1 D; CACNA1G—Calcium Voltage-Gated Channel Subunit Alpha1 G; SCN5A—Sodium Voltage-Gated Channel Alpha Subunit 5; SCN8A—Sodium Voltage-Gated Channel Alpha Subunit 8; RefSeq—Reference Sequence.

**Table 3:** Pro-invadopodia regulator and ion channel gene co-expression correlates with shorter survival in GBM patients.

Gene	Study Dataset	Number of Patients	*p*-Value	Concordance Index
CTTN	Nutt Louis [42]	50	2.12 × 10^−^^5^	65.39
CTTN+CACNA1F	Nutt Louis [42]	50	4.49 × 10^−^^6^	67.25
MMP2	Freije Nelson GPL96 [43]	85	5.95 × 10^−^^3^	59.74
MMP2+CACNA1B	Freije Nelson GPL96 [43]	85	1.71 × 10^−^^3^	62.46
MMP2+CACNA1F	Freije Nelson GPL96 [43]	85	5.05 × 10^−^^3^	60.94
MMP2+CACNA1S	Freije Nelson GPL96 [43]	85	1.87 × 10^−^^3^	62.04
MMP2+KCNH2	Freije Nelson GPL96 [43]	85	4.28 × 10^−^^4^	64.08
MMP2+KCNJ10	Freije Nelson GPL96 [43]	85	1.32 × 10^−^^3^	61.17
MMP2+KCNN4	Freije Nelson GPL96 [43]	85	2.51 × 10^−^^3^	61.97
MMP2+SCN8A	Freije Nelson GPL96 [43]	85	1.90 × 10^−^^3^	61.26
MMP9	Freije Nelson GPL96 [43]	85	2.18 × 10^−^^3^	59.84
MMP9+CACNA1B	Freije Nelson GPL96 [43]	85	1.36 × 10^−3^	63.62
MMP9+CACNA1G	Freije Nelson GPL96 [43]	85	4.06 × 10^−5^	65.92
MMP9+KCN5A	Freije Nelson GPL96 [43]	85	1.31 × 10^−4^	63.24
Nck	Freije Nelson GPL96 [43]	85	1.05 × 10^−^^3^	62.01
Nck+CACNA1C	Freije Nelson GPL96 [43]	85	3.55 × 10^−^^4^	62.17
Nck+CACNA1G	Freije Nelson GPL96 [43]	85	5.06 × 10^−^^4^	62.94
Nck+CACNA1I	Freije Nelson GPL96 [43]	85	1.61 × 10^−^^5^	64.53
Nck+CACNA1S	Freije Nelson GPL96 [43]	85	2.50 × 10^−^^4^	63.33
Nck+KCNA5	Freije Nelson GPL96 [43]	85	4.68 × 10^−^^4^	62.1
Nck+KCNH2	Freije Nelson GPL96 [43]	85	2.27 × 10^−^^4^	63.92
Nck+KCNJ10	Freije Nelson GPL96 [43]	85	1.35 ×10^−^^4^	62.56
SH3PXD2A	Yamanaka Nishio [38]	29	3.80 × 10^−2^	77.89
SH3PXD2A+KCNA5	Yamanaka Nishio [38]	29	1.49 × 10^−^^2^	84.21
SH3PXD2A+KCNJ10	Yamanaka Nishio [38]	29	1.04 × 10^−^^3^	84.21
SH3PXD2A+SCN5A	Yamanaka Nishio [38]	29	7.27 × 10^−^^3^	85.26
Src	Lee Nelson GPL570 [40]	27	3.80 × 10^−^^2^	62.36
Src+CACNA1D	Lee Nelson GPL570 [40]	27	2.10 × 10^−^^2^	64.94
Src+CACNA1G	Lee Nelson GPL570 [40]	27	3.04 × 10^−^^2^	58.91
Src+CACNA1S	Lee Nelson GPL570 [40]	27	3.39 × 10^−^^2^	61.21
Src+KCNB1	Lee Nelson GPL570 [40]	27	3.39 × 10^−^^2^	61.78
Src+SCN8A	Lee Nelson GPL570 [40]	27	2.10 × 10^−^^2^	60.92
Src	Nutt Louis [42]	50	1.10 × 10^−^^2^	56.48
Src+CACNA1F	Nutt Louis [42]	50	4.10 × 10^−^^3^	62.49
Src+CACNA1H	Nutt Louis [42]	50	6.58 × 10^−^^3^	65.8
Src	GBM TCGA	538	5.76 × 10^−^^3^	54.65
Src+CACNA1C	GBM TCGA	538	6.60 × 10^−^^3^	54.68
Src+CACNA1G	GBM TCGA	538	7.24 × 10^−^^3^	54.67
Src+CACNA1H	GBM TCGA	538	7.75 × 10^−^^3^	54.59
Src+KCNA5	GBM TCGA	538	7.48 × 10^−^^3^	54.59
Src+KCNN4	GBM TCGA	538	7.03 × 10^−^^3^	54.82

Co-expression of pro-invadopodia regulator and ion channel genes correlates with poorer GBM patient outcome as determined by analysis of GBM-derived datasets in the SurvExpress online database for cancer gene expression (the log-rank test was used to generate the *p* value).

**Table 4:** Ion channel drugs used in this study.

Drug	Indication	Ion Channel
Amiloride hydrochloride dihydrate	Cardiovascular disease	Sodium
Ouabain	Neurological disease	Sodium
Oxcarbazepine	Neurological disease	Sodium
Primidone	Neurological disease	Sodium
Procaine hydrochloride	Neurological disease	Sodium
Zonisamide	Neurological disease	Sodium
Azelnidipine	Neurological disease	Calcium
Cinepazide maleate	Inflammation	Calcium
Diltiazem hydrochloride	Cardiovascular disease	Calcium
Econazole nitrate	Neurological disease	Calcium
Flunarizine dihydrochloride	Neurological disease	Calcium
Nicardipine hydrochloride	Neurological disease	Calcium
Nilvadipine	Cardiovascular disease	Calcium
Glimepiride	Type 2 diabetes mellitus	Potassium
Glyburide	Endocrinology	Potassium
Nateglinide	Immunology	Potassium
Quinine hydrochloride dihydrate	Cardiovascular disease	Potassium
Repaglinide	Endocrinology	Potassium
Tolbutamide	Type 2 diabetes mellitus	Potassium

The degree of cytotoxicity varied amongst the cell lines and drugs. To rank the drugs based on cytotoxic efficacy, a 20–30% threshold reduction in cell viability across the concentrations was applied. This identified the three most potent drugs: flunarizine dihydrochloride, econazole nitrate, and quinine hydrochloride dihydrate, which we further investigated.

## Supplementary Materials

The following are available online at https://www.mdpi.com/2072-6694/12/10/2888/s1, Figure S1: Cell viability profile of GBM cells (LN229, U87MG, MU41) following treatment with ion channel drugs, Table S1: Invadopodia regulator genes and ion channel genes utilized in the analyses of online gene expression GBM datasets deposited within the Oncomine^®^ and SurvExpress databases, Figure S2: The uncropped Western Blot figure, Table S2: Candidate drug predicted blood–brain barrier penetrance properties. We examined information on the three candidate drugs, flunarazine dihydrochloride, quinine hydrochloride dihydrate, and econazole nitrate present in the online database, ‘DrugBank’(go.drugbank.com), Table S3: Clinically achievable plasma levels of candidate ion channel drugs.
