# Supplementary material for: Inhibition of Radiation and Temozolomide-Induced Glioblastoma Invadopodia Activity Using Ion Channel Drugs
Source: Cancers (Basel). 2020 Oct 8;12(10):2888. doi: 10.3390/cancers12102888 (PMC7599723; doi:10.3390/cancers12102888)
Supplement: Supplementary file 1 [file cancers-12-02888-s001.pdf]

Supplementary Materials: Inhibition of Radiation and Temozolomide-Induced Glioblastoma Invadopodia Activity Using Ion Channel Drugs

Marija Dinevska, Natalia Gazibegovic, Andrew P. Morokoff, Andrew H. Kaye, Katharine J. Drummond, Theo Mantamadiotis and Stanley S. Stylli

Oncomine™ Analyses

Table 1 and 2 were generated via the analysis of gene expression datasets deposited within the Oncomine™ cancer-profiling database. The automated statistical analysis components that are in-built within the platform were used to perform logical differential expression analyses between samples. *p*-values are corrected for multiple hypothesis testing using the false discovery method by the Oncomine™ program. With regard to the definition of logical analyses, each gene assessed for differential expression using the Oncomine™ platform is conducted with a Student t-test. The Oncomine™ platform utilizes the R statistical computing package, and tests are conducted both as two-sided for differential expression analysis and as one-sided for overexpression analysis.

The Oncomine™ platform employs the following within its automated analyses.

t-Tests and *p*-values:

The most common t-Test is the two-sample t-Test, which is used to compare the means (averages) of two independent samples. The null hypothesis, which is presumed to be true, is that the two groups have the same average value. A t-Test generates a *p*-value, which indicates how likely the null hypothesis (no difference between the populations) is true. If the chance is less than 5% (*p*-value of 0.05 or less), then, by convention, the null hypothesis is rejected, and we conclude instead that there is a real, statistically significant difference between the means of the two groups.

p-values and Effect Size:

p-values measure whether the difference in means between two groups is likely to occur solely by chance. Effect Size measures the amount of difference between the groups and is reflected in the t-Test statistic.

Fold Change and *p*-values:

Fold change is a valuable complement to *p*-values to assess large absolute differences between groups of samples (or classes) measured in an analysis. When relying on *p*-value to assess differences, there can be analyses where *p*-values are very significant due to a large number of samples and low sample variability within groups, but the actual difference in the magnitude, or fold change, between groups is low.

SurvExpress Analyses

Table 3 was generated via the analysis of gene expression datasets deposited within the online cancer-profiling database, SurvExpress. Patient survival analysis can be presented using a Kaplan–Meier plot, which is the graphical representation of the survival probability versus time. It is common to represent more than one Kaplan–Meier curve in the same plot, especially when comparing a low-risk versus high-risk group. Instead of a visual inspection of these curves within the plot, a Log-rank test evaluates statistically the equality of the survival curves within the plot, and therefore, it can be defined as the difference between the observed and expected events within a group and is generated as part of the SurvExpress analyses of datasets.

Concordance Index (CI) values as generated by the SurvExpress analyses are displayed for Figure 1 and Table 3 within the manuscript. The CI is a summary indicator that estimates the probability that subjects with higher-risk prediction will experience an event at a different time to the subjects of lower risk.

CI is a generalization of the AUROC (Area Under the Receiver Operating Characteristic Curve) used in classification problems. The CI is expressed in SurvExpress as follows:

$$CI = \frac{1}{|\Omega|} \sum_{i,j \in \Omega} \left\{ \begin{matrix} 1 \; if \; r_i > r_j \\ 0 \; otherwise \end{matrix} \right\}.$$

In the above formula, r_i and r_j represent the risk predictors given by the corresponding prognostic index for subjects *i* and *j*, respectively and Ω represents all subject pairs (*i,j*) where $t_i < t_j$ and subject *i* is not censored. As in AUROC, higher CI values are associated to better prediction.

SurvExpress calculates the sensitivity and specificity using each data value in determining the cutoff values. This means that it calculates many pairs of sensitivity and specificity. The program employs a customized algorithm that decides how partitions change between risk groups or to evaluate the relation between risk groups and prognostic index. It changes the cutoff point one risk group at a time so that the *p*-value is at a minimum, and the process is repeated until no further changes are required.

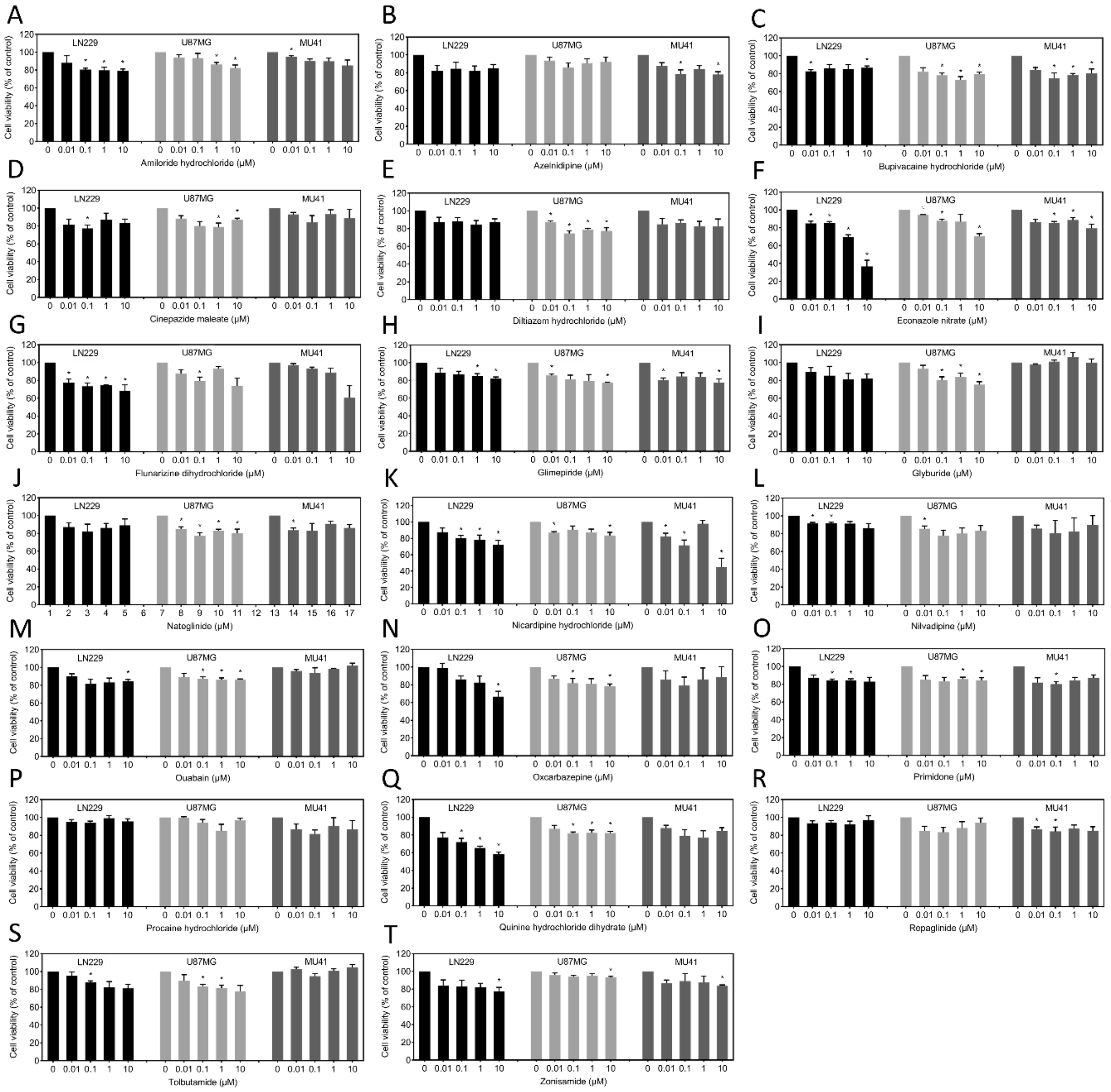

Figure S1: Cell viability profile of GBM cells (LN229, U87MG, MU41) following treatment with ion channel drugs. The GBM cells were treated with the ion channel drugs listed in Table 4 over a range of concentrations (0, 0.01, 0.1, 1, and 10 μM). Cell viability was determined using an MTT (3-(4,5-dimethylthiazol-2-yl)-2,5-diphenyltetrazolium bromide) cell proliferation assay. Cell viability is represented as a percentage relative to the control cells. Mean of $n = 3$ experiments, error bars represent SEM, $^*p < 0.05$ (relative to untreated (0 μM) control group).

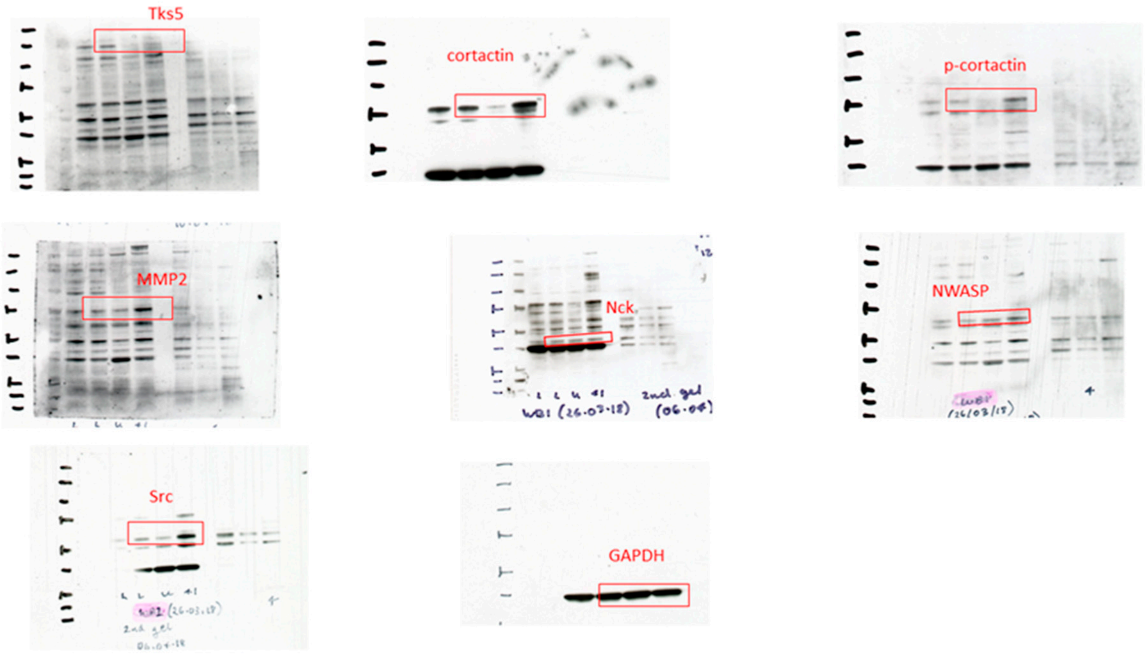

Figure 2. The uncropped western blot figure.

Table S1. Invadopodia regulator genes and ion channel genes utilized in the analyses of online gene expression GBM datasets deposited within the Oncomine™ and SurvExpress databases.

Invadopodia Regulator Genes	Ion Channel Genes
CTTN	CACNA1B
MMP2	CACNA1C
MMP9	CACNA1D
Nck1	CACNA1F
Nck2	CACNA1G
SH3PXD2A	CACNA1H
SH3PXD2B	CACNA1I
Src	CACNA1S
	KCNA5
	KCNB1
	KCNH2
	KCNJ10
	KCNN4
	SCN5A
	SCN8A

Table S2. Candidate drug predicted blood–brain barrier penetrance properties. We examined information on the three candidate drugs, flunarazine dihydrochloride, quinine hydrochloride dihydrate, and econazole nitrate present in the online database, 'DrugBank'(go.drugbank.com). DrugBank is a knowledge base consisting of clinical information such as side effects, drug interactions, and molecular-level data including chemical structures, predicted properties, and protein interactions. Based on their chemical structures, the table lists the predicted water solubility (as determined by ALOGPS) and the predicted blood–brain barrier penetrance (as determined by ADMET - Absorption, Distribution, Metabolism, Elimination, Toxicity), as extracted from the DrugBank database. A '+' ADMET value indicates a 'yes' for the predicted property of blood–brain barrier penetrance. As defined by the predicted ADMET features in DrugBank, the blood–brain barrier penetrance values of 0.9789, 0.9382, and 0.9823 indicate that there is a 97.89%, 93.82%, and 98.23% probability that flunarizine dihydrochloride, quinine hydrochloride dihydrate, and econazole nitrate will cross the blood–brain barrier. logP—drug lipophilicity; logS—drug water solubility.

Drug Name	Water Solubility ALOGPS (mg/mL)	logP ALOGPS	logS ALOGPS	ADMET Blood Brain Barrier (BBB) Penetration Level
Flunarazine dihydrochloride	0.00168	5.3	−5.4	+ / 0.9789
Quinine hydrochloride dihydrate	0.334	2.82	−3	+ / 0.9382
Econazole nitrate	0.00148	4.67	−5.4	+ / 0.9823

Table S3. Clinically achievable plasma levels of candidate ion channel drugs.

Candidate Drugs	Plasma Levels
Flunarizine dihydrochloride	580 ng/mL [1]
Quinine hydrochloride dihydrate	10 μg/mL [2]
Econazole nitrate	65 ng/mL [3]

References

1. Flunarizine dihydrochloride - Product Monograph.; AA Pharma Inc. Ontario.; Canada; July 1, 2010.
2. White, N.J.; Looareesuwan, S.; Warrell, D.A.; Warrell, M.J.; Bunnag, D.; Harinasuta, T. Quinine pharmacokinetics and toxicity in cerebral and uncomplicated falciparum malaria. *Am. J. Med.* **1982**, *73*, 564–572, doi:10.1016/0002-9343(82)90337-0.
3. Gyno-Pevaryl®, *Information professionnelle du Compendium Suisse des Médicaments®*, Janssen-Cilag AG, Zug, Switzerland, 2014. (in French)

© 2020 by the authors. Licensee MDPI, Basel, Switzerland. This article is an open access article distributed under the terms and conditions of the Creative Commons Attribution (CC BY) license (http://creativecommons.org/licenses/by/4.0/).
